# Characterization of small-to-medium head-and-face dimensions for developing respirator fit test panels and evaluating fit of filtering facepiece respirators with different faceseal design

**DOI:** 10.1371/journal.pone.0188638

**Published:** 2017-11-27

**Authors:** Yi-Chun Lin, Chen-Peng Chen

**Affiliations:** 1 Department of Public Health, College of Public Health, China Medical University, Taichung, Taiwan; 2 Department of Occupational Safety and Health, College of Public Health, China Medical University, Taichung, Taiwan; Worcester Polytechnic Institute, UNITED STATES

## Abstract

A respirator fit test panel (RFTP) with facial size distribution representative of intended users is essential to the evaluation of respirator fit for new models of respirators. In this study an anthropometric survey was conducted among youths representing respirator users in mid-Taiwan to characterize head-and-face dimensions key to RFTPs for application to small-to-medium facial features. The participants were fit-tested for three N95 masks of different facepiece design and the results compared to facial size distribution specified in the RFTPs of bivariate and principal component analysis design developed in this study to realize the influence of facial characteristics to respirator fit in relation to facepiece design. Nineteen dimensions were measured for 206 participants. In fit testing the qualitative fit test (QLFT) procedures prescribed by the U.S. Occupational Safety and Health Administration were adopted. As the results show, the bizygomatic breadth of the male and female participants were 90.1 and 90.8% of their counterparts reported for the U.S. youths (P < 0.001), respectively. Compared to the bivariate distribution, the PCA design better accommodated variation in facial contours among different respirator user groups or populations, with the RFTPs reported in this study and from literature consistently covering over 92% of the participants. Overall, the facial fit of filtering facepieces increased with increasing facial dimensions. The total percentages of the tests wherein the final maneuver being completed was “Moving head up-and-down”, “Talking” or “Bending over” in bivariate and PCA RFTPs were 13.3–61.9% and 22.9–52.8%, respectively. The respirators with a three-panel flat fold structured in the facepiece provided greater fit, particularly when the users moved heads. When the facial size distribution in a bivariate RFTP did not sufficiently represent petite facial size, the fit testing was inclined to overestimate the general fit, thus for small-to-medium facial dimensions a distinct RFTP should be considered.

## Introduction

The facial fit of respirators is crucial to how effectively the respirators may protect the users from exposure to airborne contaminants when their use is required in the workplace [1]. However, the level of facial fit that the facepiece of respirators may provide is subject to influences from a variety of factors that act independently or interactively, including the facial size of the users, the design of the facepiece, and the activity the users are engaged in when the respirators are donned [2–3]. Among these factors, facial dimensions in relation to the design of facepiece were frequently found to be of a pivotal role that dominated the inward leakage of respirators [4–8].

To ensure the proper fit of respirators to the target users, in the Europe and the U.S. new models of respirators prior to marketing are required to meet the requirements of certification [9–10], in which the facepiece of respirators is fit-tested on a panel of human subjects with facial sizes representative of the user population, conventionally being the population in the nation or of the geographical area where the respirators are designed and manufactured. The respirator fit test panel (RFTP) is typically used as a matrix for selection of candidates to serve as the representative test subjects in this process [11]. In the late 1960s, the Respirator Research and Development Section of the Los Alamos National Laboratory (LANL) developed RFTPs for fit testing based on facial anthropometrics of the personnel serving in the U.S. Air Force [12]. In the LANL endeavor two bivariate RFTPs were developed. The first RFTP was developed using the dimensions of the menton-sellion length (the face length) and the bizygomatic breadth (the face width) for fit testing full-face masks, and the second using the dimensions of the face length and lip length for fit testing half masks. In 2007, the U.S. National Institute for Occupational Safety and Health (NIOSH) updated the face length- and face width-based bivariate RFTP from a survey of 3,997 individuals in the U.S. civilian workforce [11]. The subject population was divided into three age groups: 18–29 (21.5% of the subject population), 30–44 (40.1%), and 45–66 years old (38.4%). Demographically, the subjects fell in the “White” (approximately 47%), “African American” (31%), “Hispanic” (13%), and “Other” groups (including Asians; 9%) [13]. In the NIOSH effort, a RFTP was developed from principal component analysis (PCA) of 10 head-and-face dimensions considered as being significantly related to respirator fit by the working groups on respiratory protective devices in the International Organization for Standardization. The PCA RFTP developed by the NIOSH consisted of two principal components and has served as a model in the PCA RFTP development ever since.

Facial dimensions vary considerably with ethnicity, gender, and age. Yang et al. [14] studied the deviation of 19 facial dimensions for Chinese of an age between 23 and 43 years old from both the LANL RFTPs and the NIOSH bivariate RFTP to evaluate the applicability of these existing RFTPs. The test subjects consisted of primarily university teachers and students, including both males and females (n = 461). The facial dimensions in Yang et al. were measured using a traditional method employing sliding and spreading anthropometric calipers comparable to the method applied in the LANL study. Yang et al. reported that the surveyed Chinese were of a shorter and wider facial character than the American dimensions defined in the LANL and NIOSH RFTPs. The ethnical variation in facial anthropometrics may also be significant among populations in the same geographical region. Lee and Park [15] compared the facial dimensions between Koreans and Japanese as grouped by the gender and age (20–39, 40–59, and ≥ 60 years old). Among the 23 dimensions measured and reported in Lee and Park, nine were also attempted in developing the NIOSH bivariate and PCA RFTPs, and six of them were included in the final NIOSH RFTPs, including the head breadth, interpupillary distance, face length, bigonial breadth, nose breadth, and nose height. Of these six dimensions, the Japanese population was of a size significantly greater than those determined for the Koreans in the head breadth, interpupillary distance, face length, nose breath, and nose height. Aging is another determinant to factor in when studying the change in facial contours. Zhuang et al. [16] evaluated the variation in the face shape and size among different ethnic and age groups in the U.S. based on the database originally established in developing the NIOSH bivariate RFTP [11], and observed a greater size in 13 facial dimensions for the participants of an age at least 45 years old when compared to the workers of an age of 18–29 years, with the most prominent change being in the lip length, nose breadth, and subnasale-sellion length (the nose length).

The ethnical variability in facial anthropometrics as reported in literature indicated that caution should be exercised if the existing RFTPs were to be applied in fit testing respirators for a user population different than the original by which they were developed. Chen et al. [17] evaluated the NIOSH RFTPs against the facial anthropometrics of Chinese civilian workers and found that 95% of the surveyed workers fell in the NIOSH bivariate and PCA panels but only under an uneven distribution. Two Chinese RFTPs were developed in Chen et al. following the methods adopted in developing the NIOSH bivariate and PCA RFTPs to accommodate at least 95% of the surveyed Chinese workers. In Chen et al., the anthropometric database used in developing RFTPs were originated from a large-scale, head-and-face anthropometric survey conducted for Chinese workers in 2006 [18]. In this original survey, a total of 3,000 subjects (2,026 males and 974 females) were recruited, representing geographically the central, eastern, western, south-western, and north-western China, and these subjects were sorted by age and gender. Du et al. [18] observed noticeable regional difference in the anthropometric data, with the measurements in the bitragion chin arc, face length, nose length, and nose protrusion for the workers born in the north being slightly greater than the levels observed for those born in the south.

As the fit testing provides a means to ensure the fit of respirators to their users, it has been commonly practiced in the workplace and required by the U.S. Occupational Safety and Health Administration (OSHA) as a part of the respiratory protection program in the United States. To meet the workplace demands, both qualitative fit test (QLFT) and quantitative fit test (QNFT) were recommended by the NIOSH [10,19] and promulgated in the OSHA regulation [20]. A fundamental difference between the QLFT and QNFT lies in the way the test results are delivered. In the QLFT, the olfactory or taste response of an individual to the aerosolized fit test solution (e.g., saccharin or Bitrex^TM^; the latter being a bitter-tasted solution of denatonium benzoate) was based on to reach a dichotomous decision (“pass” or “fail”). In the QNFT, the ratio of the concentration of the testing material outside the mask to that inside the mask was calculated to provide a fit factor, frequently using an ambient aerosol condensation nuclei counter [20]. This difference did not deter or discourage the use of QLFT. The OSHA suggested that when a half-mask respirator successfully passed the QLFT, the respirator was considered as of a quantitative fit factor of 100, a level required for a tight-fitting half-mask respirator to meet the requirement of facial fit.

A second difference between the QLFT and QNFT is the timing by when the test is considered a failure and terminated. In both the QLFT and QNFT, the following seven major maneuvers were exercised in sequence: normal breathing, deep breathing, turning head side-to-side, moving head up-and-down, talking, bending over, and normal breathing again. The sequence of maneuvers was arranged to resemble a stepwise screening and to realize the capability of the facepiece to accommodate increasingly complex head movements and corresponding stress. In the QLFT, the test was terminated immediately when the subject reported a taste of the testing material, regardless of the exercise the individual was engaged in. Thus, a facepiece would be considered as having passed the test only if its user completed all seven steps without sensing the solution. In the QNFT, however, in each exercise the facepiece was scored for an individual fit factor and an overall fit factor was determined by weighting in the individual factors. As a result, the situation might arise in the QNFT that the overall fit factor was considered having passed the test while the respirator failed on one or more maneuvers. While in general the QNFT exceled over the QLFT in delivering the results of fit testing in quantitative terms, it might also be associated with misinterpreting the level of facial fit as far as individual head movements were concerned [21–22]. The operation of QNFT compared to that for QLFT also required more costly equipment and training in personnel, presenting difficulty to small and medium enterprises. As the QLFT and QNFT each own unique strength and weakness, they are both considered valid and comparable methods. In practice, in the workplace the QLFT was often preferred as the primary means of fit testing [23].

### Study goals

The development of RFTPs presents a mechanism to evaluate the influence of facial dimensions to the fit of respirators and provides a platform by which the facial characteristics of different populations or user groups in relation to the faceseal of facepiece may be compared. In the current study, an anthropometric survey was conducted among the youth representing respirator users in mid-Taiwan of facial dimensions that would be considered small-to-medium according to the NIOSH [11] and Chen et al. [17] bivariate and PCA RFTPs to identify head-and-face dimensions key to developing RFTPs for application to small-to-medium facial features. A comparison was made among the RFTPs developed from this study, by the NIOSH, and by Chen et al. [17] to reveal how the differences observed in facial dimensions would impact on the grouping of facial size in RFTPs for classification of facepiece. In the third part of the study, the participants were fit-tested against three models of N95 filtering facepiece respirators of different facepiece design in accordance with the size distribution specified in the bivariate and PCA RFTPs developed from this study to examine the overall and gender-specific influences of small-to-medium facial characteristics to the fit of N95 masks commercially available in Taiwan.

## Materials and methods

### Participants and anthropometric database

In this study, a total of 206 participants (101 males and 105 females) were recruited, including the workers from Central Taiwan Science Park and industries in Taichung, Taiwan, and students attending universities in this area. The participants were of an age between 21 and 30 years old. While the age was a factor known to associate with the change in facial dimensions, in this study the age was not a screening criterion in recruiting the participants. Thus, the age distribution among the participants as described corresponded to the local workforce who might demand the use of respirators for protection in their daily duty. Candidates with prominent facial scars and diagnosed with asthmatic symptoms and cardiovascular diseases in the most recent year were excluded. Following the anthropometric survey, a total of 141 participants (70 males and 71 females) of the survey participants agreed to and participated in the fit test for three models of N95 masks. The other 65 participants did not complete the QLFT on all three models of respirators due to the bitter taste of the testing solution Bitrex^TM^, the confinement of the test hood used in the QLFT, or personal reasons. As the QLFT data from these participants were incomplete, they were excluded from further analysis for the effect of facial dimensions on respirator fit under influence of facepiece design. In this study, a proportion of the subjects participating in the anthropometric survey were invited to attend a parallel study that examined the consistency between the QLFT and the QNFT in support of using the sequential maneuvers as the basis of a ranking system in evaluating respirator fit. They were fit-tested on the same models of respirators using both fit test methods. The subjects participating in this study consisted of 27 of the 141 participants who completed the QLFT for all three models of respirators (12 males and 15 females) and an additional 59 participants who completed the QLFT for two models (29 males and 30 females). No beards were allowed for the male participants to prevent interference with fit testing. The participants in this study were briefed on the purpose, design, and experimental procedures of the study; written informed consent was obtained from all individual participants. The research protocol and the informed consent form were reviewed and approved by the Research Ethics Committee of China Medical University and Hospital (CMUH102-REC3-006). The experiments conducted in the study complied with the current laws and ethical practices in Taiwan. All procedures performed were in accordance with the 1964 Helsinki Declaration and its later amendments or comparable ethical standards.

**Fig 1:**
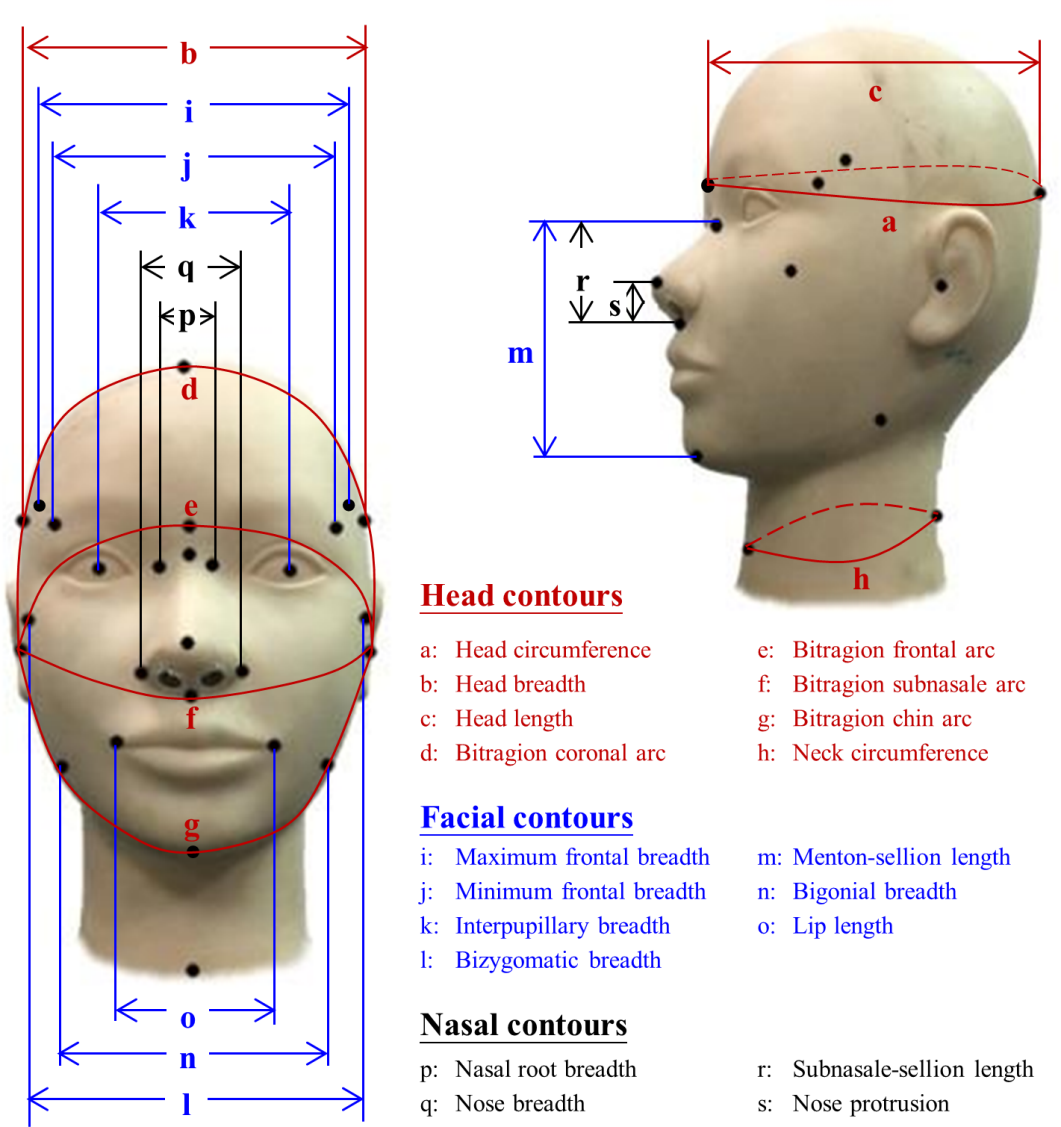
Head-and-face dimensions developed from connecting landmark points for formulation of respirator fit test panels.

The facial dimensions were measured following the protocol developed and standardized by the NIOSH [24]. Twenty-six points on the head and face were landmarked with an eyeliner pencil, and 19 dimensions in relation to the contours of the head, face, and nose were then developed by connecting sets of points and measuring the distances in between (Fig 1). These dimensions included those that were required for developing the NIOSH bivariate and PCA RFTPs for both the half and full facepieces. The facial anthropometrics were determined using the GPM Swiss Made anthropometric measurement matrix (GPM Instrument, Zurich, Switzerland) consisting of a steel measuring tape, sliding caliper, and spreading caliper. In addition, a Supore Digital PD-6 meter (Shanghai Supore Instruments, Shanghai, China) was used to measure the interpupillary distance.

### Respirator fit test panels

#### Bivariate distribution approach

The bivariate RFTP as developed by the LANL and the NIOSH adopted the face length and face width as the vectors in constructing the bivariate matrix for facial size distribution [11–12]. In these bivariate RFTPs, the minimum of the face length and face width was the mean of the values measured for females minus two standard deviations (SDs) and the maximum the mean of the values for males plus two SDs. In the bivariate RFTP, at least 95% of the population being represented was included within the panel boundaries. For selecting candidates of facial size that sufficiently covered the target population, a bivariate RFTP was typically divided into ten size cells in accordance with the method described in Zhuang et al. [11], and the cell boundaries were adjusted to distribute the population among the cells as uniformly as possible. These procedures were followed in this study for the development of bivariate RFTP based on the results of anthropometric survey.

#### Principal component analysis approach

In statistics the PCA decreases the dimensionality of a dataset consisting of many interrelated variables [25]. Compared to the bivariate distribution, the PCA technique when applied in the RFTP development allows for a three-dimensional description of facial anthropometrics that considers the stereospecific impact of facial features to the fit of respirators. The facial dimensions selected in developing the NIOSH PCA panel, as described in Zhuang et al. [11], included the head breadth, minimum frontal breadth, interpupillary distance, face width, face length, bigonial breadth, nasal root breadth, nose breadth, nose length and nose protrusion. The same dimensions were adopted in the development of PCA RFTP in Chen et al. [17]. In the NIOSH approach, the first two principal components (PCs) from the analysis were used to support the development of PCA RFTP in accordance to the eigenvectors calculated from the data. This approach was followed in this study. In addition, the procedures established in Zhuang et al. [11] were adopted to divide the PCA panel into eight cells and for assignment of the participants into individual cells.

### Facial fit of respirators of different facepiece design

#### Filtering facepiece respirators

**Fig 2:**
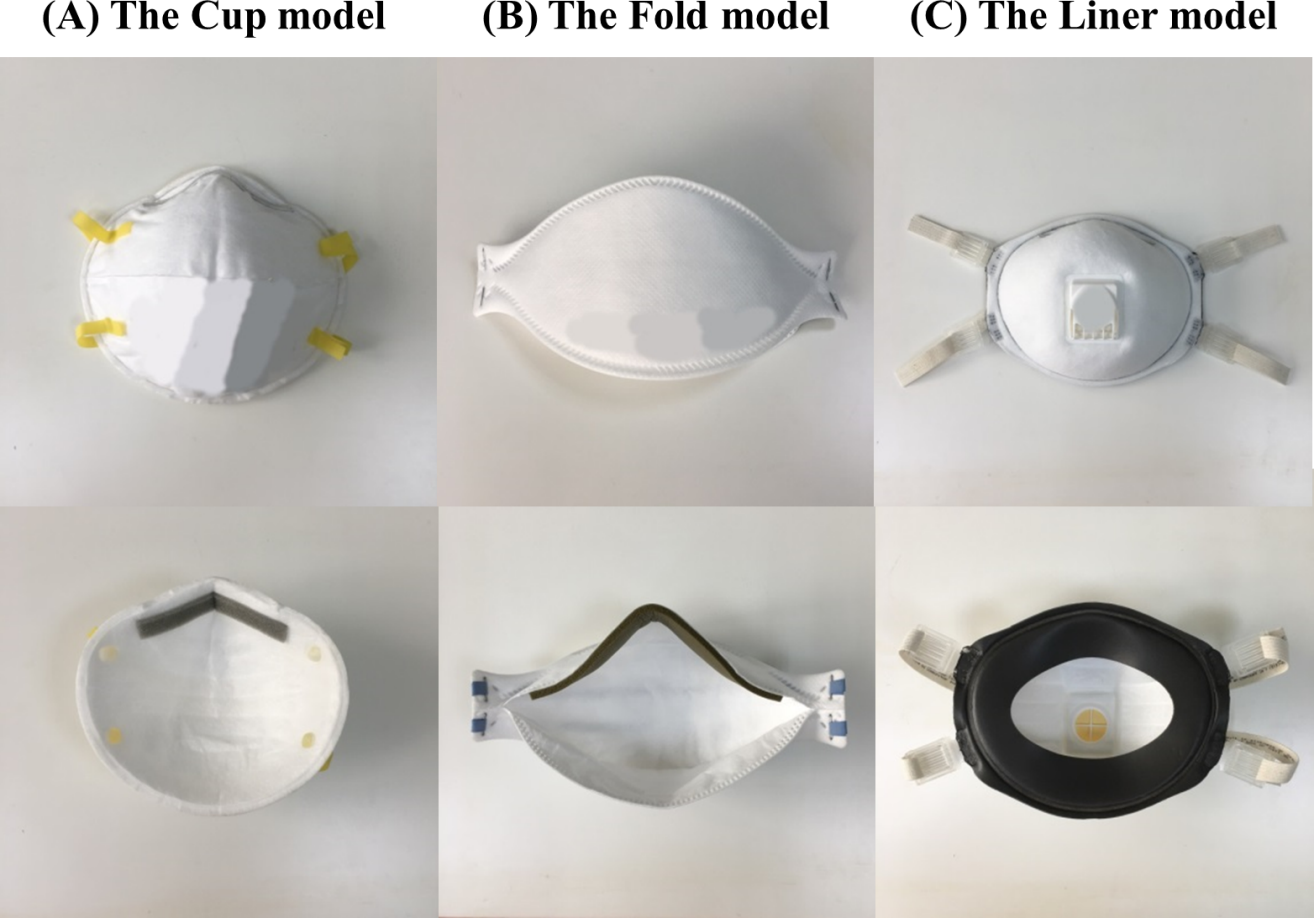
Filtering facepiece respirators of distinct facepiece design included in this study.

Three models of N95 masks with different facepiece design as available on the market in Taiwan were included in the evaluation. The first model was of a conventional design with the facepiece resembling the shape of an un-foldable, hemispherical cup (inner dimensions of 12.0 × 13.5 × 4.5 cm taken as the greatest distance in length × width × depth; hereafter referred to as “the Cup model”). The second model was a facepiece made of a three-panel flat fold (inner dimensions of 9.3 × 13.5 × 5.0 cm when the respirator was inflated and worn; “the Fold model”) that provided flexibility in shape adjustment for ease of storage and carrying. The third model was of a shape similar to the Cup model but was linered with an elastomeric faceseal band around the rim inside the mask (2.0 cm in width) and equipped with an exhalation valve for fast release of metabolic heat (inner dimensions of 10.5 × 13.5 × 4.5 cm; “the Liner model”). Fig 2 shows the appearance and facepiece design of the three models of respirators selected in this study. Of these three models, the Fold and Liner models were marketed as of a single, standard size. For the Cup model, the same facepiece design manufactured at a smaller size was available on the market but under a different model number. In this study, the standard size was selected for study as this specific model was widely used in the healthcare facilities in Taiwan as a one-size-fit-all model. In addition, using a single, standard size in the fit test provided a consistent instrument by which the effect of facial dimensions to the facepiece fit could be compared.

#### Fit testing

In the respirator fit evaluation the participants were each fit-tested against all three models of respirators. To simulate the fit test commonly practiced in small and medium enterprises in Taiwan, the QLFT procedures prescribed by the U.S. Occupational Safety and Health Administration (OSHA) in 29 CFR Part 1910.134 [20] were employed. Prior to taking the test, the participants were required to receive training and be individually evaluated for the use of respirators (donning and doffing). The participants were also supervised to perform fit check to ensure that the respirators were appropriately donned.

The QLFT consisted of two components: the sensitivity test and the actual fit test. The sensitivity test was a taste threshold screening performed prior to the actual fit test to ensure that the participants could sense the testing solution and to identify the minimum duration needed to generate and spray aerosols from the solution. In the sensitivity test the participants did not wear any respirators. In both the sensitivity and actual fit test, the TSI Qfit^TM^ automated pump-driven respirator fit tester (TSI Incorporated, Shoreview, MN, USA) was used in place of manual nebulizers to facilitate a consistent and controlled release of aerosols. The dilute solution of denatonium benzoate (Bitrex^TM^ Solutions Sensitivity and Fit Test, TSI Incorporated, Shoreview, MN, USA) was used as the base solution for generating the required aerosols [19]. The concentration of denatonium benzoate in the sensitivity solution was 12.5% of that used in the actual fit test as prescribed in the OSHA standard.

In the sensitivity test, the Qfit^TM^ was programmed to disperse in cycles of 6 sec. When the test began, each participant was sprayed with an initial dispersion of 2 cycles (12 sec) of the solution [26]. If the participant tasted the spray, the test ended. An additional dispersion of two cycles continued if the first dispersion was not sensed. A maximum of three dispersions of 6 cycles was allowed. If the subject could not taste the aerosol dispersion after 6 cycles, the teste failed. When the sensitivity test failed, the QLFT was either terminated or conducted only if the participant passed the sensitivity test at a later time after recovering from the taste fatigue. For the subjects participating in the respirator fit evaluation, they all completed and passed the sensitivity test (n = 141). The results of the sensitivity test were tabulated in the supplementary data file “S2 Dataset”. All these participants passed the sensitivity test after the initial dispersion.

Once the participant passed the sensitivity test, the actual fit test began. The initial dispersion of the fit testing solution was of the same number of cycles required to pass the sensitivity test. After the first dispersion, the aerosols were sprayed every 30 sec at a number of cycles half of the first dispersion. During the fit test, the participant performed the sequence of seven independent maneuvers in sequence as introduced earlier, with each maneuver being exercised for one minute. If the Bitrex^TM^ was tasted during the exercise of any maneuvers, the test failed and a respirator leakage was identified in association with the specific maneuver. The intermittent dispersion of aerosols continued until the fit test ended when the participant completed all seven maneuvers or when the test was terminated early in the event of a failure.

#### Evaluation of qualitative fit test results

To associate the respirator fit with the facial dimensions of the participants, the results of qualitative fit test were categorized and distributed by the facial dimensions in the individual cells of the bivariate and PCA RFTPs as well as by the respirator facepiece design. Based on the maneuvers exercised in the test, seven categories were established, including “None”, “Normal breathing”, “Deep breathing”, “Turning head side-to-side”, “Moving head up-and-down”, “Talking”, and “Bending over”. Each category was named by the final maneuver completed in the QLFT. For example, the category “Moving head up-and-down” consisted of the tests successfully completing the maneuver “Moving head up-and-down” but failing the next maneuver “Talking”. Since the resistance of facepiece to stress from head movement strengthened as the number of maneuvers being completed increased, this categorization allowed for an examination on the percentage distribution of failed tests in each stage of the QLFT and on the general trend of facial fit change under influence of facial dimensions and facepiece design. The final maneuver “Normal breathing again” was not included in the comparison as in the QLFT this maneuver was to return the series of exercises to a resting status rather than to continue an increase in facial tension for the facepiece. In practice, all participants passed “Bending over” successfully completed this final maneuver. The category “None” denotes the tests that failed the maneuver “Normal breathing” in the QLFT. To compare the facial fit among different facial sizes in the bivariate and PCA RFTPs or between different facepiece designs, the total percentage of the tests wherein the final maneuver being completed was “Moving head up-and-down”, “Talking” or “Bending over” has been selected as a threshold, as completing the maneuver “Moving head up-and-down” marks a completion for the majority of the maneuvers in the QLFT.

## Results

### Head-and-face anthropometric survey

Table 1 shows the distribution of 19 head-and-face dimensions of the subjects participating in this study as well as a comparison of these measurements to those determined by the NIOSH for the U.S. population of an age between 18 and 29 years. For the participants in this study, gender-specific difference was observed in all 19 dimensions (P < 0.001). The female dimensions were 84.7–96.5% of their male counterparts, with the most prominent difference being observed in, in the order of ascending female-to-male ratio of dimension, the neck circumference, nasal root breadth, nose breadth, bitragion chin arc, and bigonial breadth.

**Table 1:** Head-and-face dimensions of participants in this study and comparison of measurements to dimensions determined by the U.S. **National Institute for Occupational Safety and Health (NIOSH) for the U.S. population.**^a^.

Dimension	The current study			Ratio of measurement from the current study to level reported by NIOSH^b^	
	Male (n = 101)	Female (n = 105)	Ratio of female to male (%)^c^	Male (n = 551)	Female (n = 191)
Age (years)	21–30				
Height (cm)	173.9 ± 6.2	160.5 ± 4.9	92.3**	99.7	98.8**
Weight (kg)	68.5 ± 11.2	54.0 ± 8.3	78.8**	79.7**	77.9**
**Head (mm)**					
Head circumference	588.2 ± 17.6	560.0 ± 18.9	95.2**	102.7**	100.7*
Head breadth	160.5 ± 7.1	154.6 ± 7.1	96.3**	105.5**	105.4**
Head length	186.4 ± 7.2	176.0 ± 6.4	94.4**	95.0**	93.8**
Bitragion coronal arc	369.5 ± 14.6	356.4 ± 15.7	96.5**	105.3**	104.3**
Bitragion frontal arc	305.6 ± 12.6	286.1 ± 11.7	93.6**	101.1*	98.9*
Bitragion subnasale arc	296.4 ± 13.3	276.8 ± 10.5	93.4**	101.0*	99.2*
Bitragion chin arc	322.9 ± 15.5	297.4 ± 11.7	92.1**	98.6*	98.1**
Neck circumference	378.3 ± 28.5	320.5 ± 22.6	84.7**	95.8**	98.3*
**Face (mm)**					
Maximum frontal breadth	124.6 ± 6.1	118.2 ± 5.0	94.9**	111.4**	109.2**
Minimum frontal breadth	111.4 ± 5.8	107.0 ± 6.2	96.1**	105.8**	103.0**
Interpupillary distance	61.6 ± 3.5	58.8 ± 2.5	95.4**	96.6**	95.0**
Bizygomatic breadth	128.2 ± 6.6	122.7 ± 7.0	95.7**	90.1**	90.8**
Menton-sellion length	115.8 ± 6.7	107.8 ± 6.2	93.1**	95.8**	95.5**
Bigonial breadth	114.0 ± 8.8	105.5 ± 7.9	92.5**	95.8**	97.0**
Lip length	48.0 ± 4.6	44.9 ± 4.8	93.5**	96.2**	96.1**
**Nose (mm)**					
Nasal root breadth	19.3 ± 2.7	17.6 ± 2.8	91.6**	115.2**	106.8**
Nose breadth	36.5 ± 3.0	33.6 ± 2.4	92.1**	100.2	101.5*
Subnasale-sellion length	48.7 ± 3.9	45.8 ± 3.7	94.1**	96.0**	96.7**
Nose protrusion	19.8 ± 2.4	18.4 ± 2.3	93.2**	93.7**	93.0**

Dimension was expressed as mean ± standard deviation except in distribution of age for the participants in this study.

^a^ Facial dimensions reported by the NIOSH [24] were those reported for the subjects categorized in the age group of 18 to 29 years old.

^b^ One-sample *t* test was applied to test statistical significance of difference between values reported for the participants in this study and those for the U.S. population.

^c^ Independent *t* test was applied for comparison between genders.

* P < 0.05.

** P < 0.001.

Between the young participants from this study and the American subjects of a similar age reported by the NIOSH [24], all of the comparable head-and-face dimensions were different at a statistically significant level (P < 0.05), except in the male nose breadth. The dimensions of the maximum frontal breadth for males and females in this study were noticeably greater than their counterparts reported for the U.S. population, with the ratio of the value for our participants to that for Americans being 111.4 and 109.2%, respectively. The nasal root breadth for young males in this study was also considerably greater than the level measured for young American males, with the ratio being 115.2%. However the difference was less pronounced between the females of the two groups. In contrast, the face widths of the males and females in this study were of a size significantly smaller than those reported for the U.S. population, with the ratio of the level for our participants to that for the American population being 90.1 and 90.8%, respectively.

### Distribution of facial dimensions for youths of small-to-medium facial features in existing RFTPs

**Fig 3:**
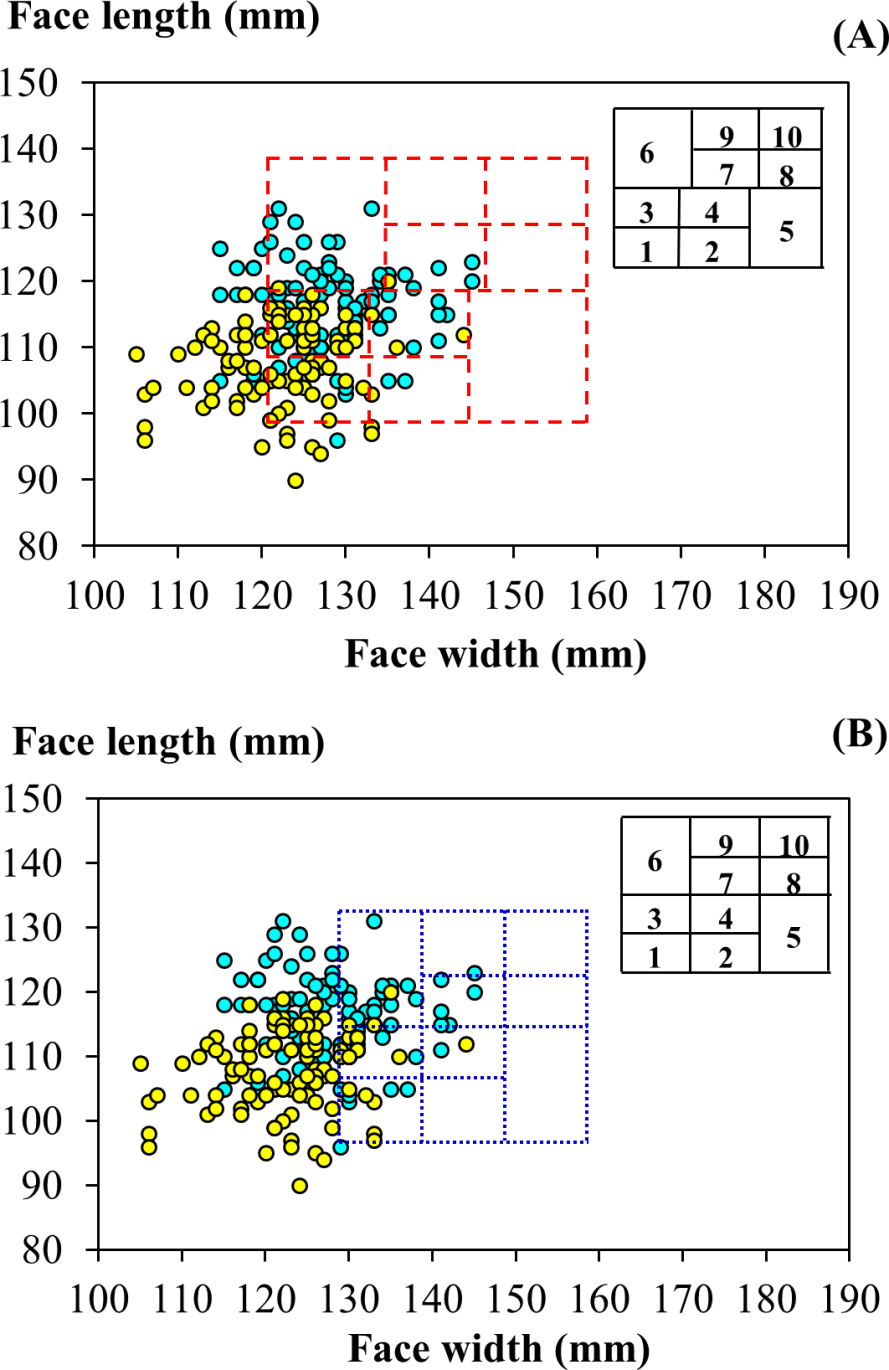
Facial dimensions of participants from this study in existing panels of bivariate design. (A) Distribution of dimensions overlaid on the respirator fit test panel developed by the U.S. National Institute for Occupational Safety and Health [11]. (B) Distribution overlaid on the panel developed by Chen et al. [17]. The cyan and yellow circles indicate values determined for male and female participants, respectively.

Fig 3 shows the distribution of face length by face width of the participants in this study in the bivariate RFTPs developed by the NIOSH [11] and by Chen et al. [17]. For the participants from this study, the face length ranged from 90 to 131 mm and the face width from 105 to 145 mm. Overall, 73.8% of the participants had facial dimensions sitting in the NIOSH bivariate RFTP, and these dimensions were concentrated in the left-lower corner of the panel. Specifically, 61.2% fell into Cells 1, 3, and 6 of the NIOSH RFTP. The bivariate RFTP reported by Chen et al. covered 32.5% of the participants in this study (47.5% of the male and 18.1% of the female participants). Approximately 88% of the included participants were distributed in Cells 1, 3, and 6 of the Chen et al. panel, the cells accommodating persons of a narrow face width.

**Fig 4:**
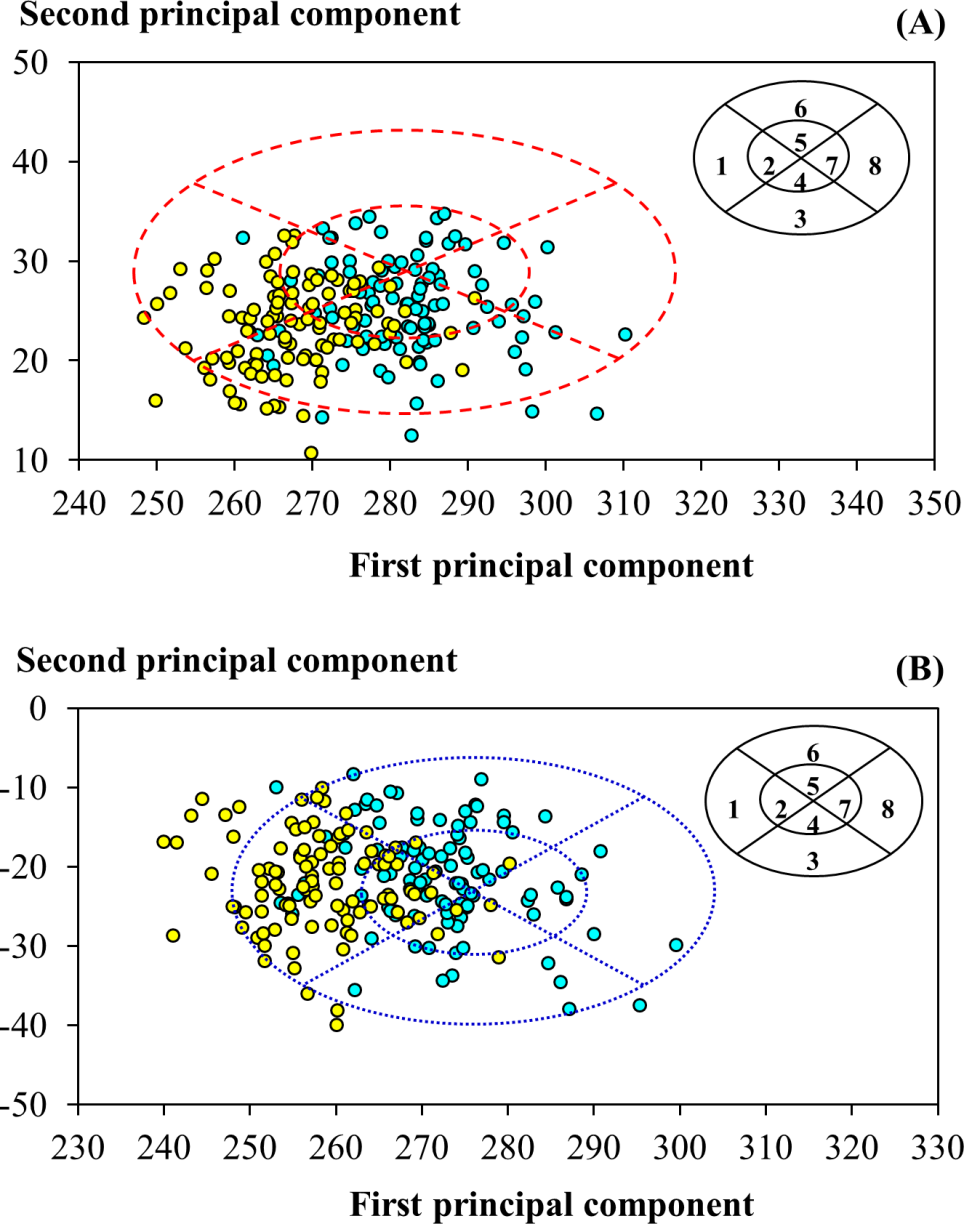
Head-and-face dimensions of participants from this study in panels of principal component analysis design. (A) Distribution of components overlaid on the respirator fit test panel developed by the U.S. National Institute for Occupational Safety and Health [11]. (B) Distribution overlaid on the panel developed by Chen et al. [17]. The cyan and yellow circles indicate values determined for male and female participants, respectively.

The PCA RFTP by the NIOSH covered overall 92.2% of the participants, including 96.0 and 88.6% of the male and female participants in this study; and the PCA RFTP by Chen et al. covered 92.2% of all participants, including 98.0 and 86.7% of the males and females (Fig 4). Similar to the earlier observation with the bivariate distribution, the females from this study were covered less by both of the existing PCA RFTPs. While the participants from this study were included in both PCA panels at a comparable level, their distributions varied. As shown in Fig 4A, in the NIOSH PCA RFTP the participants were predominantly distributed in Cells 1, 2, 3, and 4 (76.7%), with each accommodating at least 15% of the subjects. The females were more evenly distributed among these cells than the males were—each of the four aforementioned cells was occupied with at least 11.4% of the female participants whereas in Cell 1 there were only 3.0% of the male subjects. In comparison, in the PCA RFTP by Chen et al. (Fig 4B), the participants were condensed in Cells 1, 2, and 6 (67.0%), with the greatest proportion being observed in Cell 1 (35.9%). Gender-wise, the females were highly concentrated in cells that represented small facial size, Cell 1 (60.0%) and Cell 2 (17.1%), whereas the males were more evenly distributed into Cells 1, 2, 4, 5, and 6.

### Respirator fit test panels for youths of small-to-medium facial dimensions

**Fig 5:**
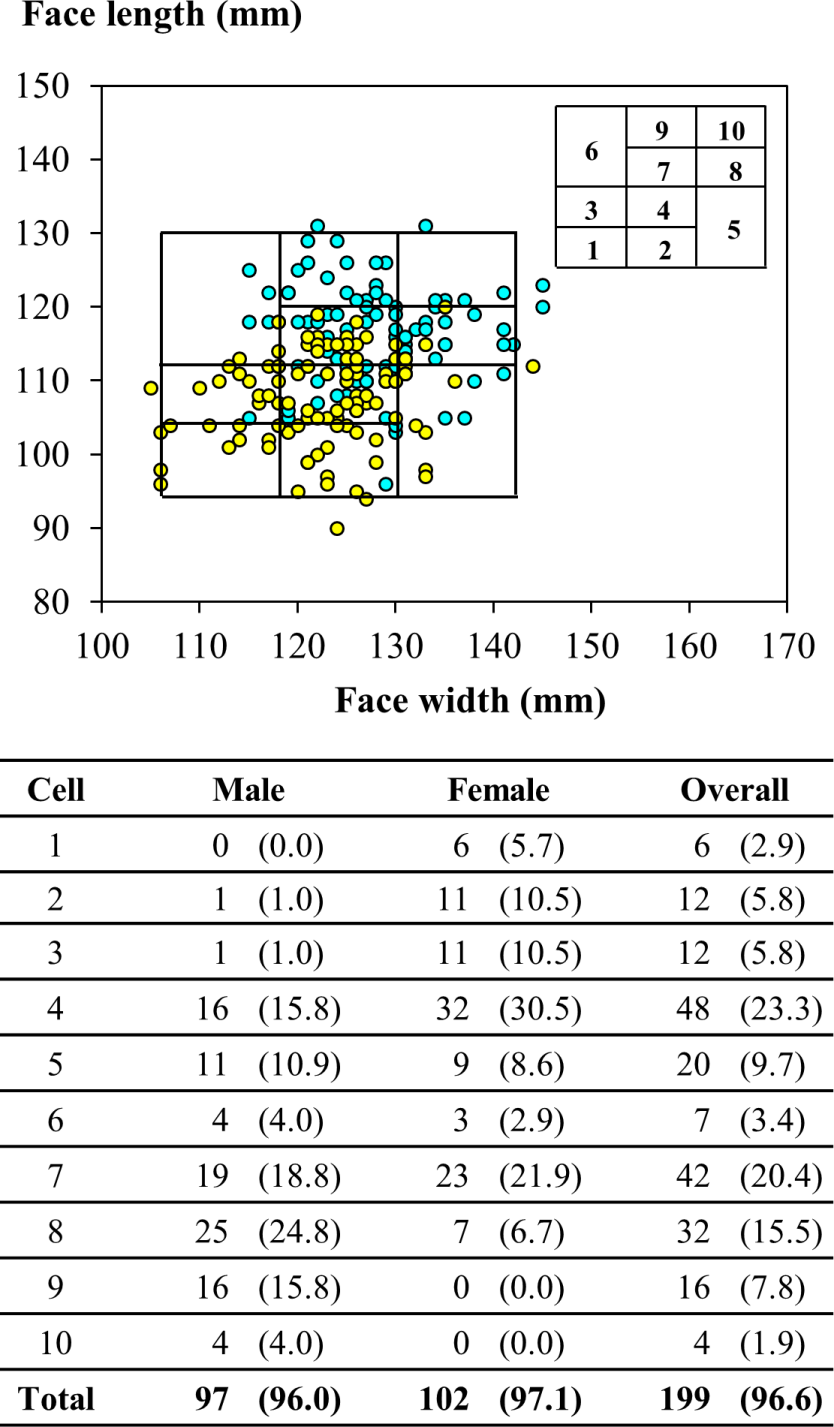
Facial dimensions of participants in panel of bivariate design developed this study. Below the scatter plot of distribution are the numbers (percentages) of participants from this study in individual cells of respirator fit test panel. The cyan and yellow circles indicate values determined for male and female participants, respectively.

**Fig 6:**
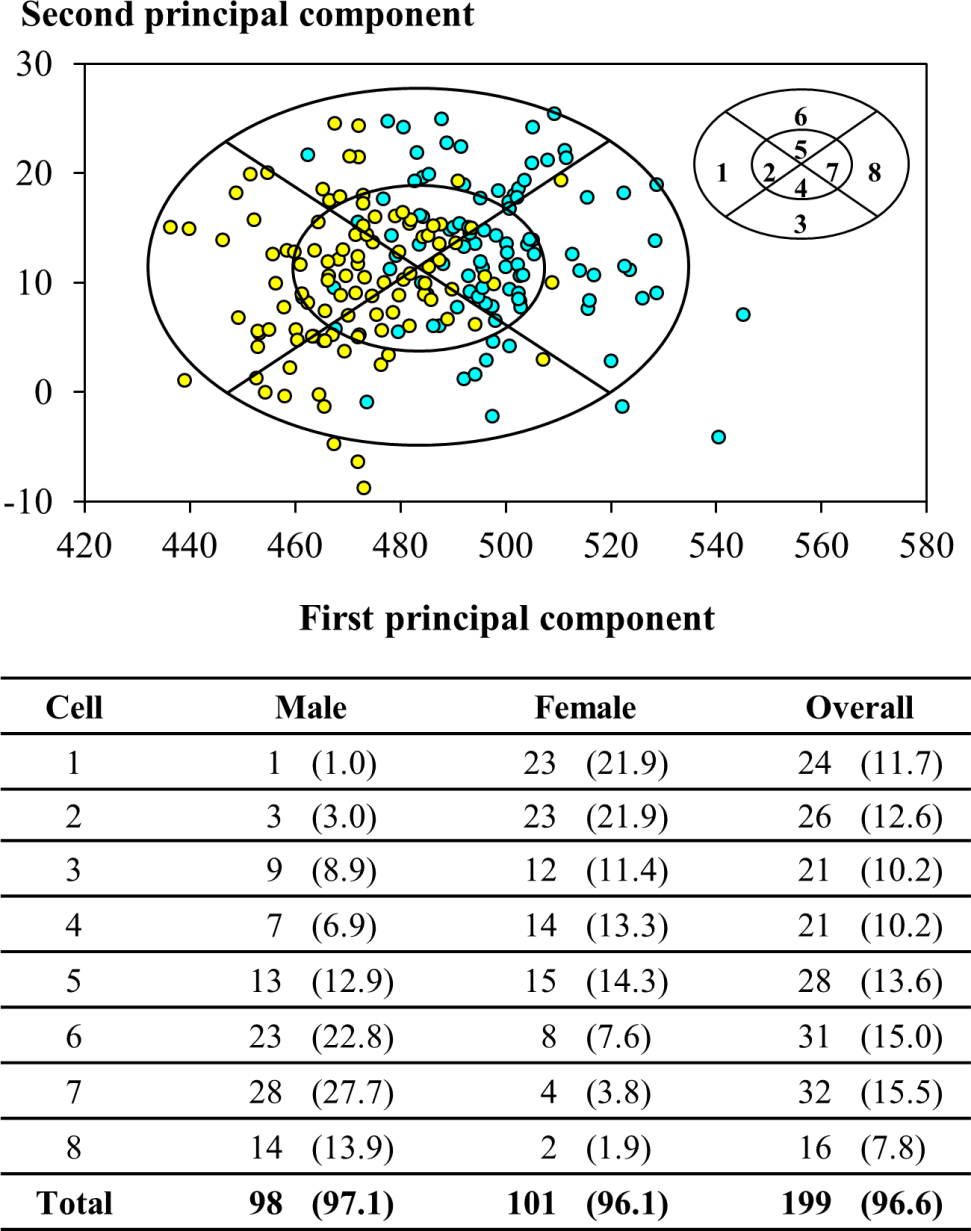
Head-and-face dimensions of participants in panel of principal component analysis design from this study. Below the scatter plot of distribution are the numbers (percentages) of participants from this study in individual cells of respirator fit test panel. The cyan and yellow circles indicate values determined for male and female participants, respectively.

Figs 5 and 6 show respectively the bivariate and PCA RFTPs developed for the participants in this study based on the results of anthropometric survey. For the bivariate panel, the face length ranged between 94 to 130 mm and the face width between 106 to 142 mm. Up to 96.6% of the participants were included in the panel, including 96.0 and 97.1% of the male and female participants, respectively. A total of 23.3 and 20.4% of the participants fell into Cells 4 and 7, the cells for medium facial size in this panel.

The statistics of principal component analysis weighted to the participants in this study were summarized in S1 Table. The PCA panel developed in this study yielded two principal components (PCs) that explained 36.6% and 12.5% of the total variance. The first PC (PC1) and second PC (PC2) each consisting of 10 head-and-face dimensions were mathematically expressed as the following polynomial equations:

$$\begin{array}{l} \mathrm{PC}1=\\ 0.641031\times HB+0.555771\times MFB+0.766379\times IB+0.665253\times \\ FW+0.644739\times FL+0.513814\times BB+0.569534\times NRB+\\ 0.712228\times NB+0.426506\times NL+0.469558\times NP \end{array}$$ (1)

$$\begin{array}{l} \;\mathrm{PC}2=\\ 0.032379\times HB–0.434911\times MFB–0.309213\times IB–0.084469\times FW+\\ 0.526096\times FL–0.060759\times BB–0.294401\times NRB–0.071652\times NB+\\ 0.724967\times NL+0.246291\times NP \end{array}$$ (2)

where *HB* was the head breadth, *MFB* the minimum frontal breadth, *IB* the interpupillary distance, *FW* the face width, *FL* the face length, *BB* the bigonial breadth, *NRB* the nasal root breadth, *NB* the nose breadth, *NL* the nose length, and *NP* the nose protrusion. All dimensions were in a unit of mm; but the principal components were unitless. The eigenvectors (coefficients) for the dimensions in PC1 were all positive; the top three were associated with facial dimensions describing miscellaneous widths of the facial contours, including the interpupillary distance, nose breadth, and face width. In contrast, in PC2 only for the nose length, face length, nose protrusion, and head breadth the eigenvectors were positive. The PCA RFTP developed for the respirator users of small-to-medium facial dimensions covered 96.6% of the participants in this study. Overall, the participants were evenly distributed in the cells (7.8 to 15.5% each). However, the females were condensed more heavily in Cells 1 to 5 (82.8%) and the males in Cells 5 to 8 (77.3%).

### Fit of N95 filtering facepiece respirators under influence of facial dimensions

**Fig 7:**
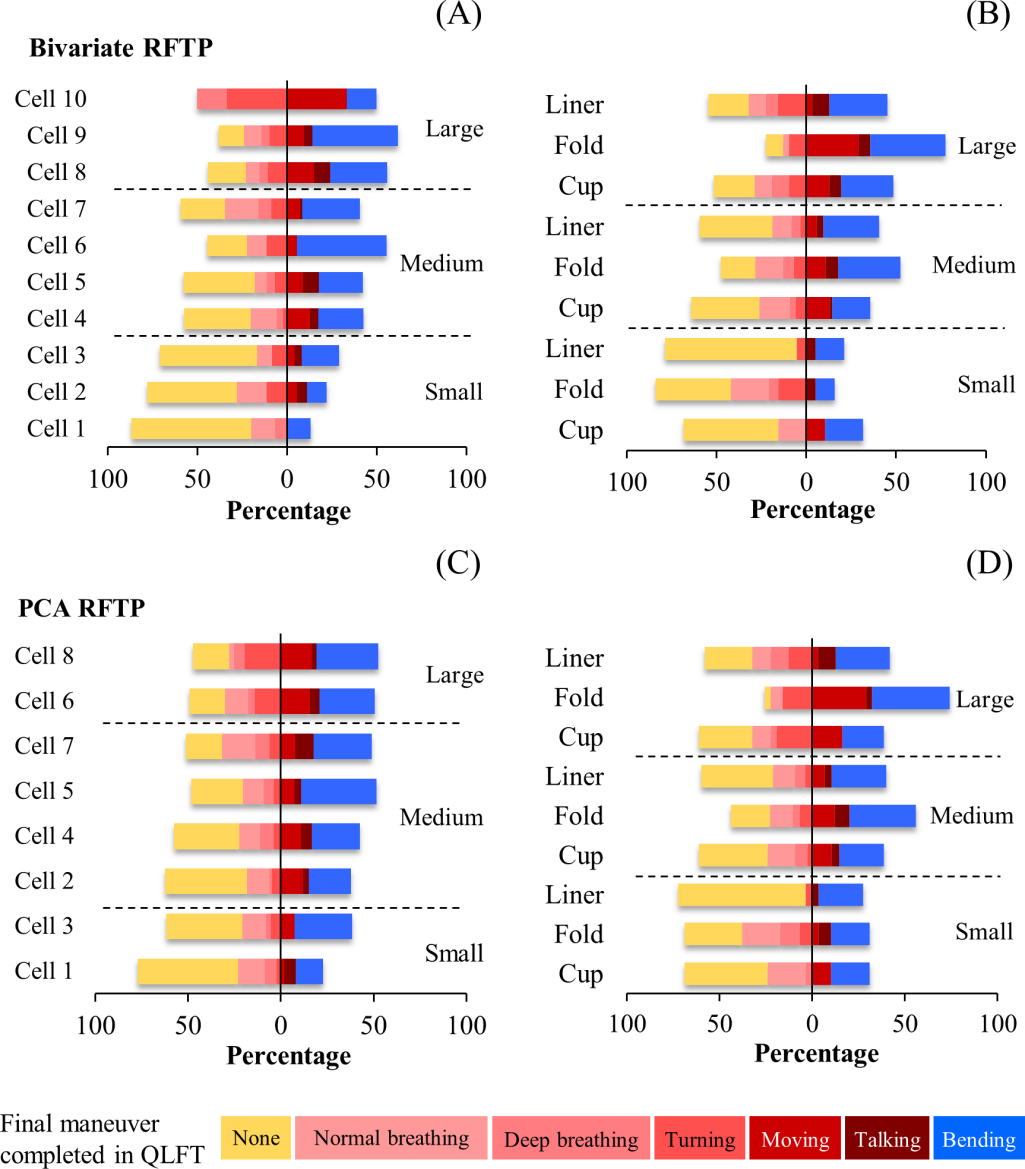
Final maneuver completed in fit test of three N95 filtering facepiece respirators. (A) Percentage of the final maneuver completed in qualitative fit test (QLFT) as distributed by cells in respirator fit test panel (RFTP) of bivariate design. (B) Percentage of the final maneuver as distributed by models of respirator and by facial sizes in RFTP of bivariate design. (C) Percentage of the final maneuver as distributed by cells in RFTP of principal component analysis (PCA) design. (D) Percentage of the final maneuver as distributed by models of respirator and by facial sizes in RFTP of PCA design. The tests categorized in the group “Normal breathing”, “Deep breathing”, “Turning” (turning head side-to-side), “Moving” (moving head up-and-down), “Talking”, or “Bending” (bending over) were those wherein the participants passed the named maneuver but failed on the next one. The group “None” denotes the tests that the participants failed on the first maneuver.

Fig 7 shows the facial fit of the three N95 filtering facepiece respirators to the participants as distributed by the cells in the bivariate/PCA RFTPs or by the facial sizes in these panels. The facial fit was indicated by the final maneuver completed in the QLFT as well as by the percentage of tests corresponding to that specific maneuver category. In the figure, the cells in the bivariate and the PCA RFTPs were partitioned in accordance with the scheme the NIOSH developed and recommended for fit testing respirators of a three-size system [11]. In the three-size system, the small, medium, and large facial size groups in the bivariate RFTP consist of Cells 1–3, Cells 4–7, and Cells 8–10, respectively, and in the PCA RFTP include Cells 1 and 3, Cells 2, 4, 5, and 7, and Cells 6 and 8.

Overall, the respirator fit increased with increasing facial dimensions among the participants. In the bivariate RFTP, the total percentages of the tests wherein the final maneuver being completed was “Moving head up-and-down”, “Talking”, or “Bending over” for the medium size group (40.7–55.6%; consisting of 59.6% of the participants) and for the large size group (50.0–61.9%; 22.0% of the participants) were significantly greater than the level for the small size group (13.3–29.2%; 13.5% of the participants), attesting to the influence of the face length and face width to the facepiece fit. The fit of the three models of filtering facepieces varied in response to the facial size change. The total percentage of the tests passing the threshold for the Fold, Cup, and Liner, models was 52.5, 37.6, and 38.3%, respectively. In the medium and large size groups, the Fold model (Medium: 52.3%; Large: 77.4%) in general fit better than the Cup model (Medium: 35.7%; Large: 48.4%) and the Liner model (Medium: 40.5%; Large: 45.2%).

In the PCA RFTP, while the trend of a rising facial fit continued as the facial size increased, the rise did not conform consistently to the size division in the three-size system. As shown in Fig 7C, the total percentage of the tests wherein the final maneuver being completed was “Moving head up-and-down”, “Talking” or “Bending over” in the medium size group varied significantly (37.9–51.9%; consisting of 53.2% of the participants) while its counterpart in the large size group (50.9–52.8%; 22.0% of the participants) in comparison was consistent. If the medium facial size group was sub-divided into small-medium and medium-large, consisting of Cells 2 and 4 and Cells 5 and 7, respectively, then a stable increase in the total percentage of tests passing the threshold along with the size change would be observed (37.9–42.6% and 49.0–51.9% for the small-medium and the medium-large groups, respectively). As far as the impact on respirator fit was concerned, the facial characteristics in the small-medium size group were more related to those in the small size group and the characteristics in the medium-large size group more to those in the large size group—the total percentage of tests passing the threshold in Cells 1–4 ranged between 22.9 and 42.6% (48.9% of the participants) and the level in Cells 5–8 between 49.0 and 52.8% (46.8% of the participants). Among the three different models of half masks, once again, in the medium and large facial size groups, the Fold model (Medium: 56.0%; Large: 74.2%) fit better than the Cup model (Medium: 38.7%; Large: 38.7%) and the Liner model (Medium: 40.0%; Large: 41.9%).

## Discussion

Fit testing respirators on human subjects selected from a RTFP consisting of various facial sizes has long been in practice as a safeguard to ensure that new models of respirators provide adequate faceseal to the target users. However, the efficacy of this practice depends on how well the RFTP represents the intended users. While different techniques were attempted in developing RFTPs that better characterized facial characteristics, little was examined on the effect of the head-and-face dimensions that varied significantly from the sizes represented in the existing RFTPs to the composition and development of RFTPs. This study developed bivariate and PCA RFTPs for respirator users of head-and-face dimensions that would be considered small-to-medium by the RFTPs currently in existence representing large-scale, regional populations. Through this process the facial dimensions key to the development of RFTPs for small-to-medium facial features and to the facial size grouping in these panels were also identified.

### Factors to consider and balance in development of RFTPs

An RFTP serves to provide a reference of facial dimensions for a population or a respirator user group by which new models of respirators may be designed or tested for facial fit. Thus, the development of a new RFTP may be required when the existing RFTPs cannot provide candidates that sufficiently represent the target respirator users. Typically, ethnical or geographical variation that translates into difference in facial dimensions is a factor when deciding if a distinctive RFTP is needed. However, even at a local scale or among a population of the same ethnicity the requirement for a new RFTP may arise—the facial characteristics among a target group of respirator users in an area do not necessarily correspond to those in the general population as defined by ethnicity, nationality, or geographical division. While these conventional divisions may serve as a starting point in consideration of a new RFTP, they are not the only criteria to reply upon in reaching a decision.

In this study, the facial dimensions from the participants were significantly smaller than those reported in the existing RFTPs. The facial size distribution in the bivariate RFTPs reported in literature were not able to sufficiently represent the petite facial sizes observed in this study, and as a result the fit testing overestimated the general fit of a mask. These observations led us to developing distinctive RFTPs for small-to-medium facial dimensions. As our participants were mostly the workers in manufacturing industries and to a lesser extent the students attending college in the Taichung area, their head-and-face anthropometrics did not necessarily correspond to the ethnical or geographical distribution of the general population in Taiwan. The RFTPs developed in this study aimed to describe the facial characteristics of the respirator users or potential users in the area rather than to represent the entire Taiwan population. In developing these RFTPs, we have also demonstrated that the RFTPs for a target group of respirator users, such as those of small-to-medium facial features included in this study, might be significantly different from the RFTPs developed for a general population. The appropriateness of applying an ethnically or geographically based RFTP to specific groups of respirator users that are of distinctive anthropometrics (e.g., age distribution) may require evaluation before the fit testing takes place as a part of workplace risk management program. Nonetheless, it is also important to balance between the development of a new RFTP and the potential scale of application—an RFTP with a narrow scale may require frequent update due to the fast change in the anthropometrics of a small population.

While the age was often a required consideration in the development of RFTP, the gender was a different concern. The gender-specific difference was consistently observed in different anthropometric measurements. However, in the RFTP development the gender as a factor has been incorporated in the panel design. This accommodation was manifested in the design of facial size grouping in the RFTP as well as in the paradigm of candidate selection for fit test. For the latter, when evaluating the facial fit of new respirators 25 candidates are required in accordance with the facial sizes distributed in a RFTP, with approximately half of the candidates being male and half female. Despite the difference in facial size, in our study both the males and females were of similar facial characteristics as suggested in the female-to-male ratio of the head-and-face dimensions presented in Table 1 (91.6–96.5). Thus, a further division of RFTPs by the gender would not be essential in our study, unless the target population was primarily female-based.

Ethnicity as a factor is typically considered when significant differences were identified in facial dimensions among racial groups. Geographically, a significant variation in anthropometrics is often investigated at this scale of a continental or regional distribution. For example, Yang et al. [27] studied the fractal characteristic of facial anthropometrics for the youths born in central China and concluded that new respiratory protective devices were needed for Chinese youths born in different geographical areas. In our survey, the ethnic distribution among Taiwanese was not considered in the RFTP development. The prevalence and transmission patterns of diseases were identified to be associated with variation in the migratory history, community distribution, or socioeconomic status of the major ethnic groups in Taiwan [28–31]. However, it was unclear if the ethnic composition among Taiwanese would be a factor influencing a local RFTP, as to our best knowledge a significant variation among ethnic groups in Taiwan was not yet confirmed in the face width and face length, the most critical facial dimensions underlying the RFTPs.

### Facial characteristics of participants in this study and distribution in existing RFTPs

**Fig 8:**
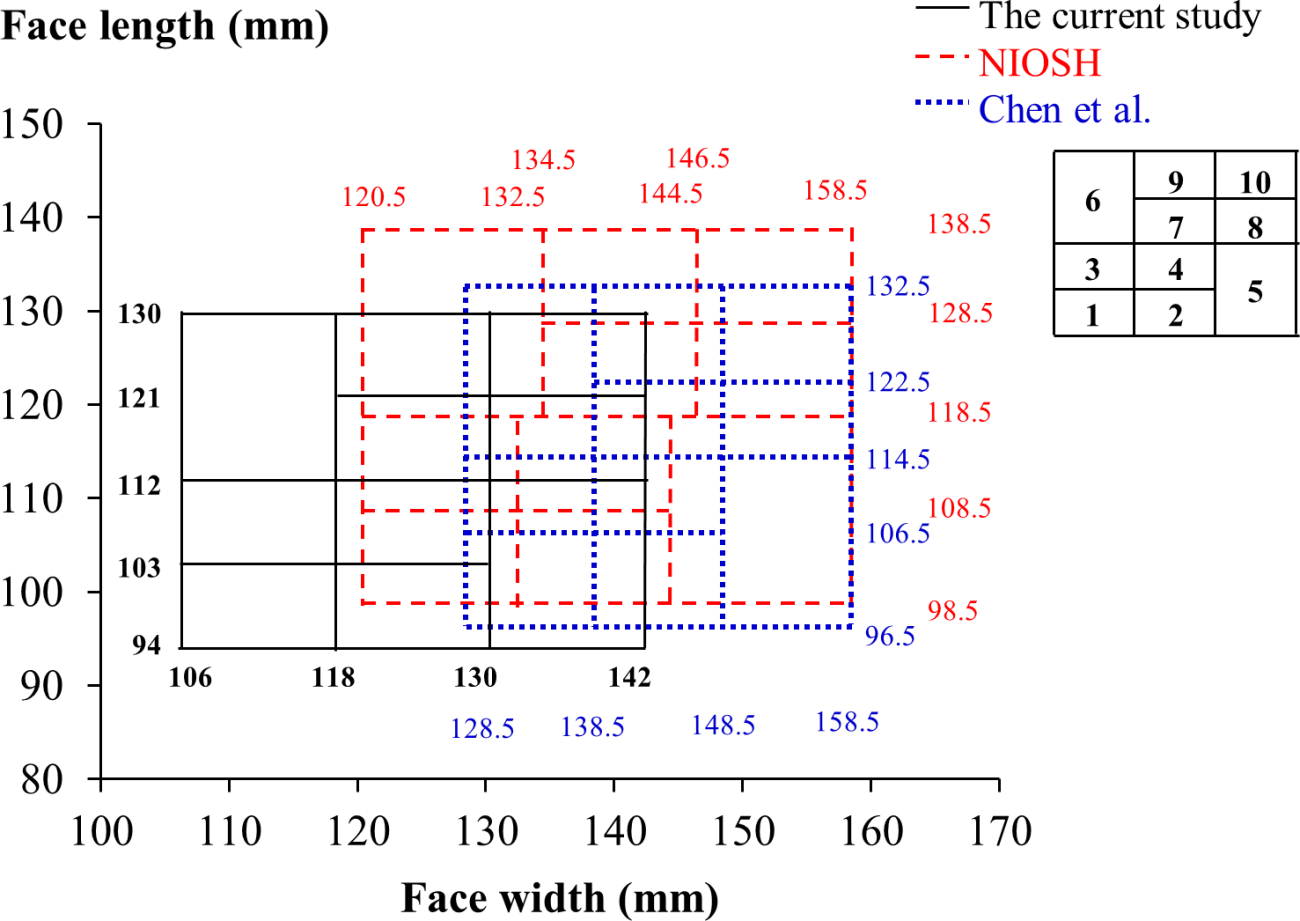
Comparison of bivariate respirator fit test panels and cell divisions. The panels compared include those developed from this study, by the U.S. National Institute for Occupational Safety and Health (NIOSH) [11], and by Chen et al. [17].

In this study, the anthropometric data were compared to those reported by the NIOSH [11] and Chen et al. [17] to quantify the deviation in head-and-face dimensions between the participants of these studies and of the current study. Between the participants in this study and the U.S. subjects, the bizygomatic breadth (the face width) presented a prominent contrast. In the bivariate RFTP, the distribution of face width by face length was employed in sizing the respirators by the facial dimensions in the three-size system. The ranges of facial dimensions specified in the bivariate RFTPs by the NIOSH and Chen et al. accommodated a high percentage of the face length measured for the young participants in this study (94.7 and 96.6%, respectively) but less of the face width (77.7 and 33.0%). The low percentage of coverage for the face width of the participants in the bivariate panel reported by Chen et al. was largely attributed to the smaller range of face width as well as the greater level in the lower bound of this range as compared to their counterparts in the NIOSH panel (Fig 8).

In this study, the female participants were of a smaller facial size than the male, and thus were covered less in either the bivariate or PCA RFTPs developed by the NIOSH and Chen et al. In the NIOSH PCA RFTP, the male participants from this study were predominantly being in Cells 2, 3, 4 and 5 and the female in Cells 1, 2, 3 and 4. These findings suggested that if the NIOSH PCA panel was to be applied in Taiwan the majority of our males would be considered as of a small-to-medium size and the females a small one. While the PCA RFTP by Chen et al. also characterized the facial size of the participants in this study as small-to-medium, the contrast between the males and the females was more prominent—the females from this study were highly concentrated in Cell 1 (60.0%), equating to a small face of narrow contours. The gender-specific difference in the coverage of the participants in the existing RFTPs further indicated the need of including and balancing both genders in the design of a RFTP.

### Respirator fit test panels developed for small-to-medium facial features

For the bivariate RFTP developed from this study, approximately 57% of the females were located in the lower-left four cells, reflecting the trait of a short-and-narrow face, whereas over 63% of the males were concentrated in the upper-right four cells symbolizing a relatively long-and-wide face. As Fig 8 shows, the bivariate RFTPs developed in this study and by Chen et al. shared both a similar range in the face length and the upper/lower bounds that deviated more from the levels identified in the NIOSH RFTP. In comparison, the bivariate panel from this study had a lower bound in the face width that was 14.5 and 22.5 mm less than its counterparts in the RFTPs developed by the NIOSH and Chen et al., respectively. In the PCA RFTP, the PC1 in our analysis essentially captured the overall width of the face, whereas the PC2 emphasized on features related to the face length as well as to the nasal contours. As a result, the facial contours that scored higher in PC2 would include long face length, prominent nasal ridge, and narrow nose wings. Oestenstad et al. [4] indicated that the leakage of half-mask respirators was strongly affected by the leaks around the nose and chin area. The PCA RFTP developed for the participants in this study was able to capture the key facial features that were associated with facial fit of respirators.

As our results show, the face width-related dimensions are a major source of variability and thus of uncertainty among the PCA RFTPs developed targeting different populations. As the magnitudes of the eigenvectors in Table 2 indicated, the three dimensions in PC1 that contributed the most to the PC1 score in the NIOSH PCA RFTP were the face width, bigonial breadth, and head breadth and for the Chen et al. RFTP were the face width, head breadth, and interpupillary distance. The only facial dimension that these two panels and the PCA panel from this study shared among the top three dimensions in PC1 was the face width. In contrast, in PC2 all three panels shared the same top three dimensions contributing to the PC score, including the face length, nose length, and nose protrusion.

**Table 2:** Eigenvectors in support of respirator fit test panels of principal component analysis design developed from this study, by the U.S. **National Institute for Occupational Safety and Health (NIOSH), and by Chen et al.**

Head-and-face dimension ^a^	First principal component			Second principal component		
	This study	NIOSH	Chen et al.	This study	NIOSH	Chen et al.
Head breadth	0.641031	0.372241	0.373045	0.032379	0.013306	−0.132683
Minimum frontal breadth	0.555771	0.343264	0.322260	−0.434911	−0.152951	−0.388836
Interpupillary distance	0.766379	0.363474	0.370307	−0.309213	−0.173099	−0.159748
Bizygomatic breadth (face width)	0.665253	0.426498	0.422051	−0.084469	−0.039087	−0.140757
Menton-sellion length (face length)	0.644739	0.329648	0.244826	0.526096	0.359799	0.568632
Bigonial breadth	0.513814	0.372717	0.328562	−0.060759	−0.093279	−0.227790
Nasal root breadth	0.569534	0.202311	0.159204	−0.294401	−0.341235	−0.173192
Nose breadth	0.712228	0.301125	0.321181	−0.071652	−0.210833	0.079405
Subnasale-sellion length (nose length)	0.426506	0.193650	0.297905	0.724967	0.584261	0.528574
Nose protrusion	0.469558	0.113578	0.237882	0.246291	0.551842	0.308739

^a^ Head-and-face dimensions reported by the NIOSH were described in Zhuang et al. [11] for subjects of an age distribution from 18 to 65 years old; those reported by Chen et al. [17] were for subjects from 18 to 66 years old.

In this study, only two PCs were considered for the PCA panel developed using the measured anthropometric data, as the purpose here was to compare the RFTP from this study to those reported in literature and to demonstrate in a quantitative manner the impact of change in facial dimensions on the RFTP and the facial size grouping. In this approach, the method designed by the LANL for developing the bivariate panel [12] and the method by Zhuang et al. for developing the PCA panel [11] were used. As previously described, in the PCA panel from this study the PC1 described the overall width of the face and the PC2 the features related to face length. In PC3, the prominent dimensional changes that were not observed in PC1 and PC2 included a negatively weighted subnasale-sellion length and a significantly weighted nose protrusion; both possibly would describe the influence of nasal dimensions to respirator fit for petite facial features. As insufficient information was available to describe the dimensions in relation to the median plane from the ten dimensions currently measured, an anthropometric interpretation of PC3 would be difficult.

Zhuang et al. [11] commented that four criteria should be considered in determining the number of PCs to be retained in the PCA panel, including (1) an eigenvalue of a PC greater than 1 (Kaiser criterion), (2) a significant proportion of variance to account for, (3) a substantive meaning for a retained component, and (4) practicality. The eigenvalue of the PC3 in our study was 1.057 and the cumulative percentage total variance for PC1 and PC2 was 49.149%. If the first two criteria in Zhuang et al. alone were considered, a PC3 would be included in the panel. However, in meeting the third and fourth criteria Zhuang et al. also cautioned that the PCA scores calculated from two components allowed for a two-dimensional scatter plotting of the panel for a direct comparison with the previous LANL panel and the bivariate panels. When additional PCs were retained, the scatter plots would be challenging to interpret. By the same consideration, Chen et al. also opted for a PCA panel of two components for the Chinese civilian workforce, although an eigenvalue of 1.214 was identified for PC3. As the PCA RFTP developed in the current study consisting of two PCs sufficiently covered over 96% of the participants and provided anthropometric interpretation of the fit test results comparable to those from the existing RFTPs, a three-component PCA panel was not attempted in this study.

### Facial fit of respirators in relation to division of facial size in RFTPs

**Fig 9:**
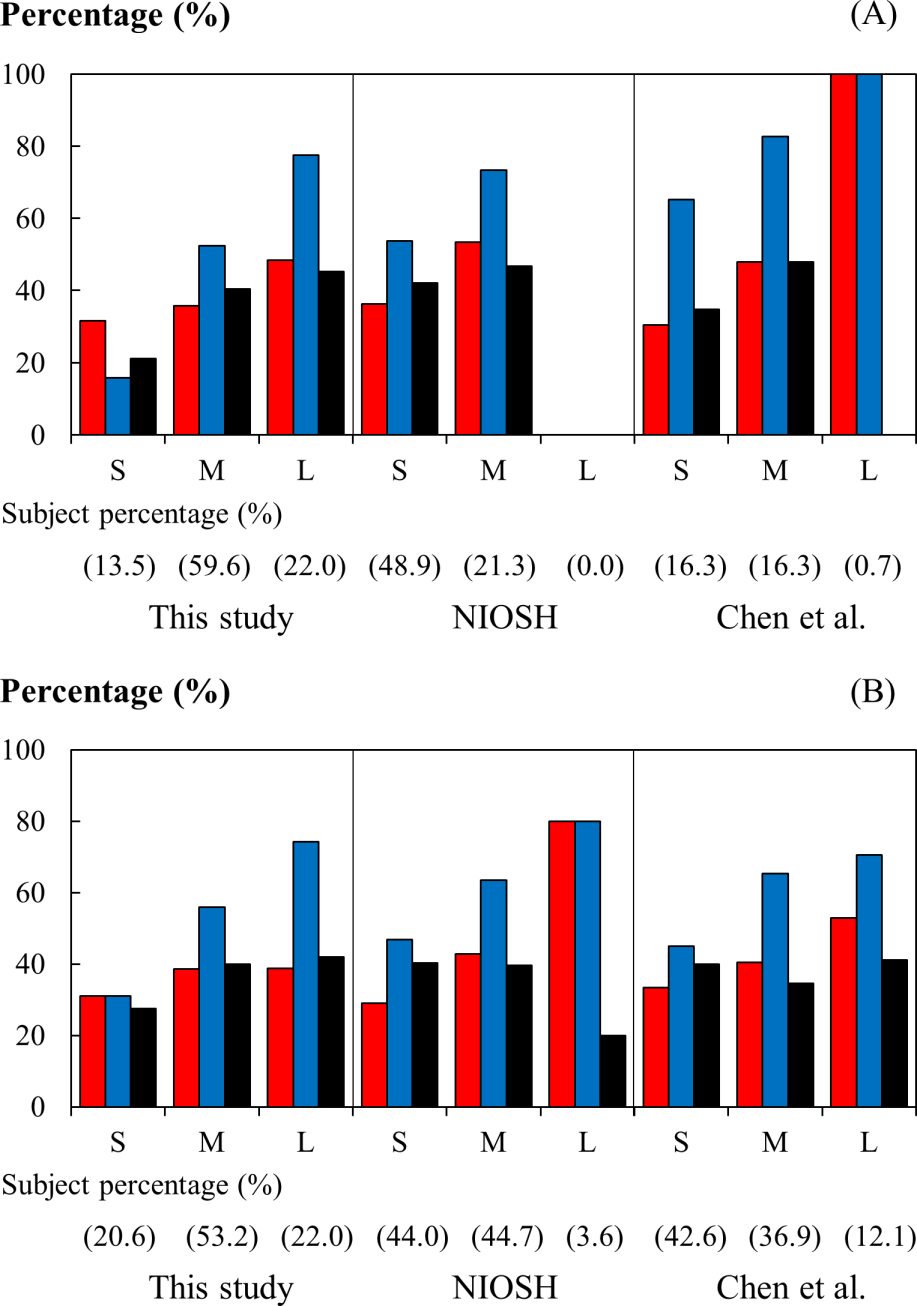
Completion of final maneuvers in qualitative fit test as distributed by facial size. (A) The total percentage of tests wherein the final maneuver being completed was “Moving head up-and-down”, ‘Talking” or “Bending over” as distributed by the facial size defined in the respirator fit test panels (RFTPs) of bivariate design developed from this study, by the U.S. National Institute for Occupational Safety and Health [11], and by Chen et al. [17]. (B) The total percentage of tests as distributed by the facial size defined in the panels of principal component analysis design. The red, blue, and black bars indicate the percentage determined for the respirators of different facepiece design, the Cup, Fold, and Liner models, respectively. The capital letters S, M, and L denote the small, medium, and large facial sizes, respectively.

For the respirator users of small-to-medium facial features, the variation in face width-related dimensions impacted on the division of facial size in the RFTPs and thus on the interpretation of results from fit testing the subjects selected in accordance with the facial size grouping. Fig 9 shows the facial fit of the three N95 masks tested in this study as distributed by the facial size groups defined in the RFTPs developed from this study, by the NIOSH, and by Chen et al. The facial fit was indicated by the total percentage of the tests wherein the final maneuver being completed was “Moving head up-and-down”, “Talking”, or “Bending over”.

Overall, when in a RFTP the facial size coverage did not sufficiently represent the petite facial dimensions of a target population, the RFTP was inclined to provide candidates that would show better facial fit in fit testing. This bias in interpreting fit testing results was pronounced in the bivariate panel but not in the PCA panel. Among the subjects participated in the fit test, the bivariate RFTP developed using the anthropometric data determined in this study accommodated 95.1% of the participants. In comparison, the NIOSH bivariate RFTP covered 70.2%, mostly in the small size group (48.9%), and the Chen et al. RFTP 33.3%. When the total percentage of tests passing the threshold for the tested facepieces in the small and medium facial size groups of different RFTPs were compared, the fit reported for the NIOSH and Chen et al. panels in most cases exceeded their counterpart reported for the panel developed in this study. An exaggeration of facial fit likely occurred when the small facial anthropometrics were misrepresented in the RFTP. Compared to the bivariate panel, the RFTP developed adopting a PCA design was more lenient to the inclusion of varying facial anthropometrics—all three PCA panels were able to accommodate at least 91.6% of the participants in the fit test and as a result the respirator fit determined for the same size group among different RFTPs was comparable. However, caution should be exercised when interpreting the results reported for the large facial size group of a PCA RFTP if the panel did not have sufficient coverage of the target population, as the total percentage of tests passing the threshold might be distorted in this group, possibly being exaggerated, due to the relatively small percentage of the subjects.

For the PCA RFTP developed to adapt to small-and-medium facial contours, we propose to modify the three-size classification system that the NIOSH suggested by dividing the original medium size group into two groups, the small-medium and medium-large groups, to better associate the change in the respirator fit with the facial size change in the panel. In the PCA RFTP developed in this study, as our results show (Fig 7), the contrast in the total percentage of tests passing the threshold between Cell 1 and Cell 8 represented the effect of the face width on the respirator fit, while the change from Cell 3 to Cell 6 symbolized the effect as the face and nose gradually lengthened. When the NIOSH designed the PCA RFTP, as the cell moved up in PC2 the nose also gradually shifted from the wide to narrow shape, as evidenced by the eigenvector determined for the nose breadth [11]. However, such a shape change was not observed in this study, as for the participants surveyed in this study the widening of the nose breadth was observed in PC1, i.e., a similar change in the nasal width would occur when the cell moved down in PC1 from Cell 8 to 1. The inclusion of nose breadth as a key dimension in PC1 of the PCA RFTP from this study rendered the PC1 in this panel more sensitive to the change in nose shape, thus a sub-division of the medium group into sub-groups would be appropriate.

### Effects of facepiece design and exercise to respirator fit

The three models of N95 masks included in this study each was of a distinct facepiece design. There might be variations within filtering facepieces of the same generic design. For examples, improvements could be made to reduce discomfort of the users by the installation of an exhalation valve, of an adjustable nose clip, or of adjustable buckle straps. However, significant difference in facial fit was identified only between facepieces of different designs [32]. Thus, the three models of filtering facepieces included in this study provided a representative coverage for the respirators of different facepiece design on the Taiwanese market. The evaluation as being discussed here was intended to provide an interpretation on the respirator fit under influence of the facepiece design, not to serve as an investigation on the efficacy of specific models of respirators fitting the users.

Among the three different facepieces, the Fold model in general fit better than the Cup and Liner models in the medium and large facial size groups, except in the total percentage of tests passing the threshold for the large size group of the bivariate RFTP by Chen et al. (Fig 9), which was likely a biased estimate as there was only one participant distributed in this category. In the Fold model, the three-flap fold allowed for greater adaption of the facepiece to the face if the mask could sufficiently cover the area, particularly when the users were engaged in activities wherein significant head movement was involved. In comparison, the Cup model adopted a conventional, rigidly shaped facepiece that was less accommodating to change in the facial size or head movement. The Liner model did not provide a better performance, even though the facepiece was attached with an elastomeric liner to enhance its faceseal. This was perhaps a result of burdening the rigid cup-shaped mold of the facepiece with an additional elastomeric liner and the consequential loss in its adaptability to facial contours and movement.

In addition to the facial dimensions and facepiece design, the exercise that the users of respirators were engaged in when donning the respirator also exerted an influence to the fit. As demonstrated in Fig 7, when the fit for the three different N95 masks was compared against the facial dimensions in the bivariate RFTP, even for the participants in the large facial size group, the total percentage of the tests failing prior to or at the maneuver “Talking” ranged from 51.6 to 64.5%, indicating that for the tested respirators the inward leaking began once the users began to move their heads. For the small and medium facial size groups, the total percentage of failing became significant as soon as the users breathed normally (50.0–66.7% in the small size group and 22.2–40.0% in the medium size group). Similarly, in the PCA RFTP for all facial sizes the QLFT failed mostly when or before the participants began to talk (55.5–79.2%), indicating an increase in stress to the head straps of respirators once the head movement began.

**Fig 10:**
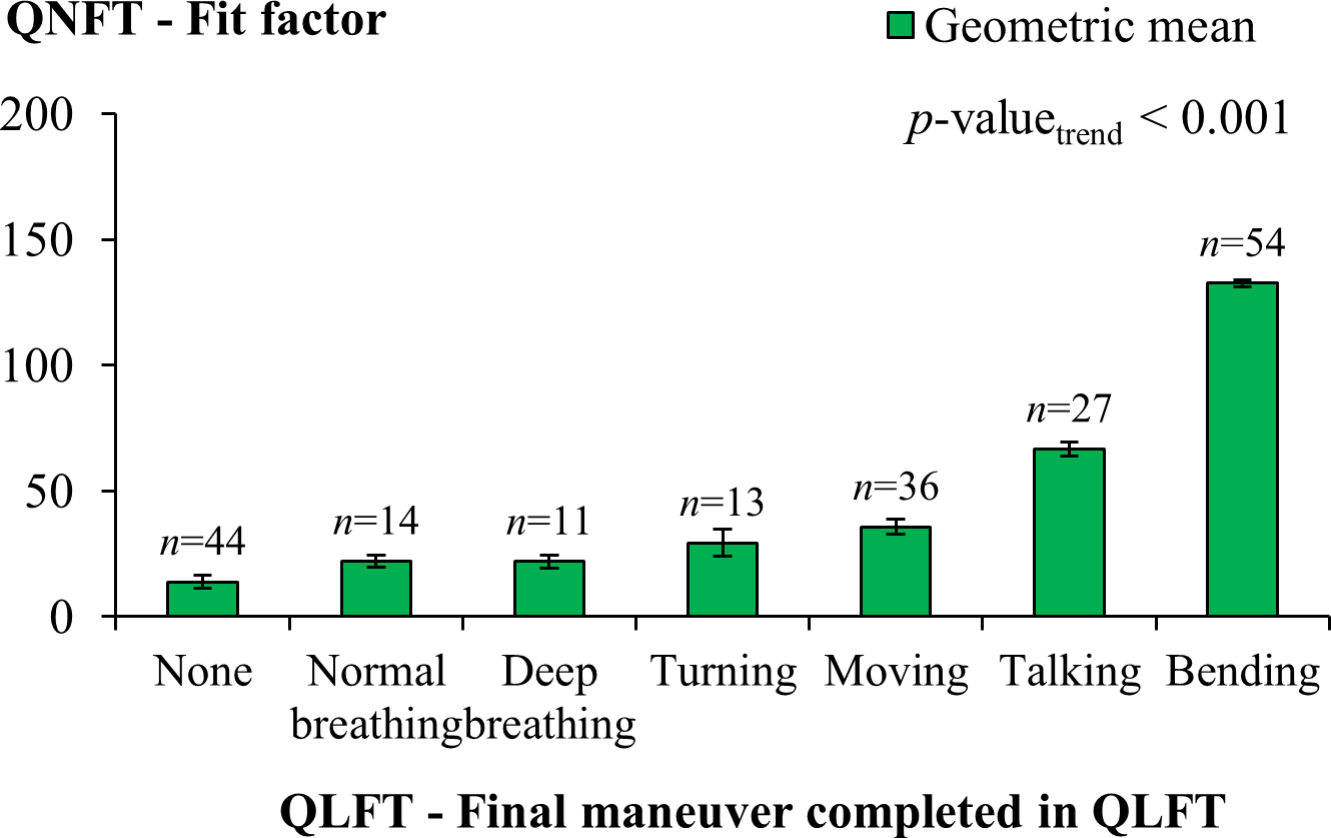
Fit factor in association with the maneuver by which qualitative fit test was terminated. The overall fit factor for a respirator was calculated from the factors reported for individual maneuvers as determined using the quantitative fit test (QNFT) distributed by the final maneuver completed in the qualitative fit test (QLFT) for the same respirator. The tests categorized in the group “Normal breathing”, “Deep breathing”, “Turning” (turning head side-to-side), “Moving” (moving head up-and-down), “Talking”, or “Bending” (bending over) were those wherein the participants passed the named maneuver but failed on the next one. The group “None” denotes the tests that the participants failed on the first maneuver.

In this study, the sequence of maneuvers exercised in the QLFT was relied upon as the basis of a ranking system to evaluate the level of facial fit under the assumption that the stress added to the facepiece increased as the sequence of maneuvers continued. An approach to evaluate this assumption was to examine the change in the fit factor of the filtering facepiece as individual maneuvers in the QLFT took place in sequence. In our study, a proportion of our participants were invited to fit test on the same models of respirators using both the QLFT and QNFT. In both the QLFT and QNFT, the same seven maneuvers were exercised following the identical sequence. A total of 199 tests performed by 86 participants were analyzed. Fig 10 shows the change in the overall fit factor of the respirator as determined by QNFT and distributed by the final maneuver completed in the QLFT for the same respirator. In the QLFT, the final maneuver “Normal breathing again” was incorporated in the sequence to allow the subject to recover from the test, not to impose additional stress. In practice, all the participants passing the maneuver “Bending over” also passed “Normal breathing again”. To envision the trend of change in the fit factor as a result of stress from head movement, the maneuver “Bending over” was considered the last step of stress challenge.

As the results show, the overall fit factor increased when more maneuvers were successfully completed in the QLFT, demonstrating that the current sequence of maneuvers when exercised was able to challenge the facepiece with increasing stress. The observed probability of concordance between the QNFT and the QLFT results was 79.4% (kappa statistic = 0.510). In the QLFTs where all the maneuvers were completed (i.e., completing “Bending” in Fig 10), a geometric mean of 132.5 for the fit factor was observed, exceeding the requirement set forth in the OSHA’s regulatory demand of 100 for half-mask facepieces. When the users in the QLFT successfully completed the step “Moving head up-and-down” but failed on “Bending over”, the fit factor ranged between 35.7 and 66.7; whereas the fit factor for the users who failed before or at the step “Moving head up-and-down” ranged from 13.8 to 29.4. These results suggested that the stress from the current sequence of head movements in the QLFT was lower in its early steps and greater later, supporting the use of these maneuvers as an indicator of facial fit for the facepiece in this study. Furthermore, the fit factor for the tests completing the maneuver “Moving head up-and-down” was identified to be statistically different from those determined for the tests stopping at earlier maneuvers. This result suggested that the maneuvers in the QLFT before and beyond the aforementioned maneuver presented distinct levels of stress to the facepiece. This result corresponds to and supports the selection of the total percentage from the tests wherein the final maneuver completed in QLFT was “Moving head up-and-down”, “Talking” or “Bending over” as a threshold of facial fit in this study.

## Conclusions

A RFTP developing from facial size distribution representative of the intended users of respirators is essential to ensure the proper fit of the respirators to their target users. In this study, an anthropometric survey was conducted among youths representing respirator users in mid-Taiwan, and the bivariate and PCA RFTPs were developed for application to small-to-medium facial features. These panels were then used in fit testing N95 masks of distinct facepiece design to realize the influence of facial characteristics on the fit of filtering facepieces. The findings from this study demonstrate the need of using a representative RFTP in the fit test of respirators if the respirators are to be used by a population of key facial dimensions at variance with those by which these respirators were designed. The PCA distribution in panel design was more amenable to varying facial anthropometrics and thus better accommodated the impact of variability in facial dimensions. When in a RFTP the facial size coverage did not sufficiently represent the petite facial size, the RFTP was inclined to provide candidates that would show better facial fit in fit testing.

## Supporting information

S1 Appendix Algorithm on classification of study participants into the principal component analysis panel cells. (DOCX)

S1 Dataset Anthropometric data including 19 head-and-face dimensions for the participants in this study. (XLSX)

S2 Dataset Data of fit testing participants in this study with three N95 filtering facepiece respirators. (XLSX)

S3 Dataset Results of qualitative and quantitative fit tests performed in parallel for respirator fit evaluation. (XLSX)

S1 Table Summary statistics for principal component analysis weighted to the participants in this study. (XLSX)
