# Supplementary material for: Characterization of small-to-medium head-and-face dimensions for developing respirator fit test panels and evaluating fit of filtering facepiece respirators with different faceseal design
Source: PLoS One. 2017 Nov 27;12(11):e0188638. doi: 10.1371/journal.pone.0188638 (PMC5703559; doi:10.1371/journal.pone.0188638)
Supplement: S1 Appendix — (DOCX) [file pone.0188638.s001.docx]

**S1 Appendix. Algorithm on classification of study participants into the principal component analysis panel cells.**

PC1 mean = 484.012020; SD = 20.109353.

PC2 mean = 11.511440; SD = 6.450230.

x = PC1 – 484.012020

y = PC2 – 11.511440

slope = 6.450230/20.109353 = 0.320758

a = 2.54 × 20.109353 = 51.077757

b = 2.54 × 6.450230 = 16.383584

c = 1.175 × 20.109353 = 23.628490

d = 1.175 × 6.450230 = 7.579020

r_1_ = sqrt((x^2^)/(a^2^)+(y^2^)/(b^2^))

r_2_ = sqrt((x^2^)/(c^2^)+(y^2^)/(d^2^))

where

x and y are new coordinates of translating the origin of PC1 and PC2 from their mean values to zero;

slope is the slope value for the two lines dividing the ellipse into eight cells;

a is the constant for the length of the semi-major axis for the outer ellipse;

b is the constant for the length of the semi-minor axis for the outer ellipse;

c is the constant for the length of the semi-major axis for the inner ellipse;

d is the constant for the length of the semi-minor axis for the inner ellipse;

r_1_ and r_2_ are calculated values to determine where a particular data point or a subject is, e.g. the data point is outside the outer ellipse when r_1_ > 1 or on the outer ellipse when r_1_ = 1 or inside the outer ellipse when r_1_ < 1.

Use the x, y and r_1_ values and the algorithm below to determine if the subject is in cells 1, 3, 6 and 8:

| x | y | r_1_ | abs(y)/abs(x) | Cell |
| --- | --- | --- | --- | --- |
| ≥ 0 | ≥ 0 | ≤ 1 | ≤ slope | 8 |
| ≥ 0 | < 0 | ≤ 1 | < slope | 8 |
| ≥ 0 | < 0 | ≤ 1 | ≥ slope | 3 |
| < 0 | < 0 | ≤ 1 | > slope | 3 |
| < 0 | < 0 | ≤ 1 | ≤ slope | 1 |
| < 0 | ≥ 0 | ≤ 1 | < slope | 1 |
| < 0 | ≥ 0 | ≤ 1 | ≥ slope | 6 |
| ≥ 0 | ≥ 0 | ≤ 1 | > slope | 6 |

If the r_2_ value is less than or equal to 1, use the following algorithm to adjust the cell number:

| Cell | r_2_ | Adjust Cell |
| --- | --- | --- |
| 8 | ≤ 1 | 7 |
| 3 | ≤ 1 | 4 |
| 1 | ≤ 1 | 2 |
| 6 | ≤ 1 | 5 |
